# Elevated serum asprosin and ANGPTL8 gene expression as novel biomarkers for the diagnosis and prognosis of acute coronary syndrome

**DOI:** 10.3389/fcvm.2025.1562234

**Published:** 2025-04-04

**Authors:** Zainulabdin Abdulnabi Abdulah Albreej, Gholamreza Dehghan, Alireza Nourazarian, Naser Aslanabadi, Jamshid Assadi

**Affiliations:** ^1^ Department of Biology, Faculty of Natural Sciences, University of Tabriz, Tabriz, Iran; ^2^ Department of Basic Medical Sciences, Khoy University of Medical Sciences, Khoy, Iran; ^3^ Cardiovascular Research Center, Tabriz University of Medical Sciences, Tabriz, Iran

**Keywords:** ACS, asprosin, ANGPTL8, biomarker validation, diagnosis, prognosis

## Abstract

**Introduction:**

Managing acute coronary syndrome (ACS) remains a major global healthcare concern. Identifying novel biomarkers is crucial to improving early detection and patient classification. Traditional markers such as cardiac troponins have limitations, including delayed detectability in blood samples, necessitating the search for better alternatives. Asprosin and angiopoietin-like protein 8 (ANGPTL8) have recently emerged as potential biomarkers for ACS diagnosis.

**Methods:**

This comparative study included 100 participants, equally divided into ACS patients and healthy controls with matched demographics. Enzyme-linked immunosorbent assay (ELISA) was employed to quantify asprosin concentrations, while real-time polymerase chain reaction (RT-PCR) assessed ANGPTL8 gene expression levels. Receiver operating characteristic (ROC) curve analysis evaluated the diagnostic utility of these biomarkers, and Spearman's correlation was used to examine relationships between variables.

**Results:**

Asprosin levels were significantly elevated in ACS patients (5.27 ± 0.67 ng/ml) compared to healthy individuals (3.82 ± 1.20 ng/ml, *P* < 0.001). ROC analysis demonstrated high diagnostic performance, with an area under the curve (AUC) of 0.95, 94% detection accuracy, and 85% precision in true negative identification. Similarly, ANGPTL8 expression was markedly increased (*P* < 0.001), showing an AUC of 0.83, 88% detection accuracy, and 64% specificity. A strong positive correlation was observed between asprosin and ANGPTL8 (*r* = 0.795, *P* < 0.0001).

**Discussion:**

The findings highlight the potential of asprosin and ANGPTL8 as promising diagnostic and prognostic markers for ACS. Their high sensitivity and correlation suggest a complementary role in early ACS detection. However, further clinical trials are required to validate these biomarkers in broader patient populations and determine their practical implementation in medical settings.

**Conclusion:**

Asprosin and ANGPTL8 exhibit strong diagnostic potential in ACS detection, potentially improving early intervention strategies. Future studies should focus on their integration into clinical practice for enhanced patient outcomes.

## 1 Introduction

Despite significant advancements in therapeutic approaches, ACS, which includes unstable angina and heart attacks, remains a major contributor to health complications and fatalities worldwide (1). Detecting ACS quickly and accurately assessing risk for optimal patient care is still challenging in clinical settings. Current diagnostic markers, such as cardiac troponins specific to the heart, are not as practical due to their delayed appearance in blood tests (2, 3). In addition, traditional inflammation-related biomarkers like C-reactive protein (CRP) and interleukin-6 (IL-6) are not accurate enough in predicting clinical outcomes (4, 5). Therefore, there is a need for more advanced diagnostic methods.

From a cellular perspective, the development of ACS involves a complex interplay of compromised metabolic functions and inflammatory mechanisms (6). Disturbed glucose and fat metabolism and local and systemic inflammation play crucial roles in forming arterial plaque and subsequent heart-related events (7). Current research has identified two promising molecular candidates: asprosin and angiopoietin-like protein 8 (ANGPTL8). Of particular interest is asprosin, a protein secreted from fat tissue that is stimulated by hunger. It helps maintain blood sugar balance through various pathways. It primarily stimulates the liver to produce glucose through cyclic AMP (cAMP) signaling and regulates food intake by communicating with brain cells (8, 9). Increased levels of asprosin are associated with metabolic disruptions, reduced insulin sensitivity, and diabetes, all of which are risk factors for heart disease (10).

Furthermore, this compound triggers inflammatory responses, mainly through the nuclear factor kappa B (NF-*κ*B) pathway, leading to blood vessel damage and inflammation (11). Similarly significant, ANGPTL8, also known as betatrophin, is a protein produced in the liver that plays a critical role in controlling fat metabolism. Its inhibitory action on lipoprotein lipase affects blood fat levels, a known factor in arterial hardening (12). Scientific evidence shows that abnormal ANGPTL8 activity is related to increased fat accumulation, widespread inflammation, and plaque buildup in blood vessels. Its involvement in immune cell movement and the formation of fatty deposits in arterial walls is particularly noteworthy (13).

While these compounds independently significantly affect metabolic and inflammatory pathways, their combined impact on ACS progression is poorly understood. Their shared activation of inflammatory cascades suggests potential cooperative effects in advancing the disease. Initial studies have indicated interactions between asprosin and ANGPTL8 in the development of arterial plaque, but further evaluation is needed to determine their potential as diagnostic and predictive tools. Therefore, this investigation addresses current research gaps by analyzing blood-based asprosin concentrations and ANGPTL8 gene activity as potential markers for ACS. By incorporating these molecular indicators into existing diagnostic systems, this research seeks to improve early identification methods and enhance risk assessment procedures, leading to better patient treatment strategies.

## 2 Material and methods

### 2.1 Study design and participants

This case-control investigation examined asprosin and ANGPTL8 as potential biomarkers for ACS, conducted with full ethical approval from Tabriz University Medical Ethics Committee and informed written consent from participants. The study population was comprised of 100 subjects, and the study was evenly divided between patients with ACS and healthy controls. Based on previous research, power analysis determined that a minimum of 45 participants per group was required to achieve 80% statistical power at the 5% significance level. However, to enhance statistical reliability and account for potential participant attrition, the final sample size was expanded to 50 participants per group, resulting in a total of 100 participants.

Patient recruitment was conducted at Shahid Madani Heart Hospital, Tabriz, encompassing adults aged between 44 and 75 with confirmed ACS diagnoses. Exclusion criteria included concurrent cardiac conditions (myocarditis and cardiomyopathy), non-cardiac pathologies (such as pulmonary embolism and pneumonia), and metabolic disorders. Patients with a history of liver disease (e.g., cirrhosis, hepatitis, or significant liver dysfunction) were excluded to prevent confounding effects on biomarker levels. Glycated hemoglobin (HbA1c) measurements confirmed diabetes exclusion below 5.7%. The control cohort, recruited through Hakim Laboratory, Tabriz, included adults over 30 without a history of cardiovascular disease and normal asprosin levels. To minimize confounding variables, cases and controls were matched for age, sex, body mass index, smoking status, and metabolic parameters. Additional variables, including physical activity patterns, dietary habits, and medication regimens, were documented using questionnaires and integrated into the statistical framework.

Study enrollment was conducted at two medical facilities in Tabriz, Iran, with clinical cases sourced from the Shahid Madani Heart Hospital and control participants from the Hakim Laboratory of Pathobiology Medical Diagnosis. The study included patients aged between 44 and 75 years who received cardiologist-verified diagnoses of either unstable angina or myocardial infarction, including both ST-segment elevation and non-elevation variants. Patients with several medical conditions were excluded: cardiac-related issues (including myocarditis, cardiomegaly, cardiac injury, and congestive heart failure), systemic illnesses (such as pulmonary embolism, pneumonia, anemia, and cerebrovascular incidents), and pre-diabetic disorders (including lipid abnormalities, endocrine system disruptions, and autoimmune pathologies). To ensure consistency in metabolic health, all participants underwent blood screening to confirm the absence of diabetes, with glycated hemoglobin measurements required to fall below 5.7%.

The control population consisted of individuals above 30 with normal asprosin concentrations and no prior cardiovascular conditions. To strengthen the validity of our comparative analysis, we implemented a matching protocol between the control subjects and cardiac patients across five essential characteristics: chronological age, sex distribution, body composition indices, tobacco use patterns, and metabolic health indicators. Additional lifestyle and health variables were documented through structured surveys that captured exercise routines, nutritional patterns, and pharmaceutical usage data. These supplementary factors were incorporated into the analytical framework to enhance our findings' robustness and minimize potential confounding influences.

### 2.2 Anthropometrics and biochemical measurements

The initial data collection, including each study participant’s demographic characteristics, medical history, and physical measurements, is presented in Table 1. Weight and height measurements were used to compute body mass index according to standard formulas. Blood pressure readings were obtained using automated devices, following a standardized rest period of 10 min. Researchers gathered additional health information through structured patient interviews and reviews of medical documentation focusing on tobacco use, genetic predisposition to heart disease, and current medications. Blood collection procedures involved morning samples from fasting participants, with all subjects maintaining a 12-h food restriction overnight. The samples were drawn into specially treated tubes containing EDTA. Laboratory processing included centrifugation at specified parameters (4,000 rpm at 4°C for 10 min) to extract the serum and plasma components. These separated samples were then divided into portions and preserved at −80°C for subsequent testing. Blood asprosin levels were measured using specialized immunoassay techniques (ZellBio GmbH ELISA system), adhering to the manufacturer's specifications. This testing method demonstrated high precision, with a detection threshold of 0.25 ng/ml and reliability measures showing variation below 5% within tests and 8% between tests. Additional blood chemistry evaluations were performed using advanced analytical equipment (Roche Cobas C311 system) to measure multiple parameters: blood sugar levels, cholesterol components (total, HDL, LDL, and triglycerides), kidney function markers (creatinine), and electrolytes (sodium, potassium).

**Table 1:** Baseline parameters of the non-ACS and ACS groups.

Parameters		Control Mean ± SD (*n* = 50)	ACS patients Mean ± SD (*n* = 50)	*P* value
Age (years)		54.60 ± 12.21	58.78 ± 8.15	0.052
Sex	Female, *n* (%)	19 (38)	17 (34)	0.678
	Male, *n* (%)	31 (62)	33 (66)	
Asprosin (ng/ml)		3.82 ± 1.20	5.27 ± 0.67	<0.001**
FBS (mg/dl)		84.20 ± 5.43	120.57 ± 40.08	<0.001**
HDL (mmol/L)		41.80 ± 3.08	41.71 ± 6.92	0.470
LDL (mmol/L)		82.60 ± 22.50	99.00 ± 22.06	0.128
Creatinine (mg/dl)		0.93 ± 0.13	1.10 ± 0.20	0.023*
Cholesterol (mg/dl)		143.10 ± 27.80	177.00 ± 33.96	0.009*
Triglyceride (mg/dl)		123.70 ± 77.62	134.14 ± 86.75	0.084
Na (meq/L)		138.09 ± 2.97	141.42 ± 2.07	<0.001**
Ca (mg/dl)		9.33 ± 0.50	9.22 ± 0.99	0.070
K (meq/L)		4.71 ± 0.49	4.34 ± 0.54	<0.001**
*P* (mg/dl)		3.94 ± 0.81	3.05 ± 0.53	<0.001**
AST (U/L)		24.40 ± 7.12	31.14 ± 19.17	0.545
ALT (U/L)		27.10 ± 13.49	22.00 ± 17.23	0.609
ALP (U/L)		220.80 ± 42.90	142.71 ± 28.97	0.146

* *p* < 0.05.

** *p* < 0.01.

### 2.3 RNA extraction and gene expression analysis

RNA was extracted from whole blood samples using TRIzol reagent (Thermo Fisher Scientific, USA) following the manufacturer's protocol. The RNA concentration and purity were assessed using a NanoDrop 2000 spectrophotometer (Thermo Fisher Scientific). Samples with an A260/A280 ratio of 1.8–2.0 were considered suitable for further analysis.

#### 2.3.1 cDNA synthesis

cDNA was synthesized from 1 μg of RNA using the RevertAid First Strand cDNA Synthesis Kit (Thermo Fisher Scientific, USA).

#### 2.3.2 Quantitative real-time PCR (RT-PCR)

The evaluation of ANGPTL8 gene expression using the real-time polymerase chain reaction technique, utilizing SYBR Green detection methods on a LightCycler 480 platform from Roche. The study standardized the data against beta-2 microglobulin (B2M), the internal reference gene. The precise DNA sequences used for amplification are listed in Table 2.

**Table 2:** Primers sequences.

Gene	Primer sequence
ANGPTL8	Forward: 5′-AACAGCCTGGGTCTCTATGG-3′
	Reverse: 5′-GTCCCGTAGCACCTTCTGT-3′
B2M	Forward: 5′-GAGTATGCCTGCCGTGTGAA-3′
	Reverse: 5′-TGCGGCATCTTCAAACCTCC-3′

RT-PCR cycling conditions were as follows: initial denaturation at 95°C for 10 min, followed by 40 cycles of denaturation at 95°C for 15 s, annealing at 60°C for 30 s, and extension at 72°C for 30 s. The relative gene expression was calculated using the 2^−ΔΔCt^ method.

### 2.4 Statistical analysis

Statistical evaluation was performed using SPSS version 25 software. The initial analysis assessed data distribution patterns using the Kolmogorov–Smirnov test. The results were presented in standard format: arithmetic means with standard deviations for normally distributed measurements and median values with interquartile ranges for non-normal distributions. Between-group statistical comparisons utilized t-tests for normal distributions and Mann–Whitney U-tests for non-parametric data. The research team evaluated the relationship between clinical measurements and biological markers using Spearman's correlation analysis. The diagnostic accuracy assessment of both asprosin levels and ANGPTL8 expression relied on ROC curves. The analysis incorporated multivariate logistic regression to account for potential interfering factors, generating odds ratios with corresponding 95% confidence intervals. Statistical significance was set at *p* < 0.05. Visual data representation was performed using GraphPad Prism version 9 software.

## 3 Results

### 3.1 Biochemical parameters analyses

We first established appropriate statistical methods based on the data distribution patterns in our investigation of the differences between cardiac patients and healthy individuals. We employed the Kolmogorov–Smirnov test to determine normality, followed by either t-tests or Mann–Whitney U-tests, as appropriate. Bonferroni correction set our significance level at *p* < 0.003 to maintain statistical rigor across multiple comparisons. The demographic analysis demonstrated balanced group characteristics. The patient cohort (*n* = 50) showed a sex distribution of 66% males and 34% females, matching the ratio of 62% males and 38% females (*p* = 0.678). Age profiles were also comparable, with patients averaging 58.78 ± 8.15 years and controls 54.60 ± 12.21 years (*p* = 0.052). Blood analysis revealed several distinctive patterns between the groups. Cardiac patients exhibited higher concentrations of asprosin (5.27 ± 0.67 vs. 3.82 ± 1.20 ng/ml, *p* < 0.001) and enhanced ANGPTL8 gene expression (*p* = 0.001). Additionally, these patients had elevated levels of fasting glucose, creatinine, cholesterol, sodium, and triglycerides (all *p* < 0.05). Notably, the potassium and phosphorus concentrations were significantly reduced in patients with cardiac disease (*p* < 0.001). Other markers, including lipoproteins (HDL and LDL), calcium, and liver enzymes (AST, ALT, and ALP), remained consistent between the groups. Since asprosin and ANGPTL8 levels are influenced by liver function, we examined liver function markers (ALT, AST, and ALP). ACS Patients and Controls were not different from each other (*P* > 0.05), Indicating no liver dysfunction and confounding biomarker measurements in the present study.

### 3.2 Correlation between serum asprosin levels and different biochemical factors

Analysis of relationships among blood markers, presented in Table 3, revealed distinct patterns between the patient and control populations. Using Spearman's rank correlation method, selected for its suitability with non-normally distributed data, we examined the associations between asprosin concentrations and other biochemical measurements. Asprosin levels were not significantly associated with other blood markers in patients with cardiac disease. However, healthy participants exhibited a significant inverse relationship, where asprosin concentrations decreased as creatinine (correlation coefficient: −0.355, *p* = 0.011) and potassium levels (correlation coefficient: −0.290, *p* = 0.041) increased. These findings highlight the potentially different regulatory mechanisms of asprosin in healthy and cardiac conditions.

**Table 3:** Relationships between serum asprosin and some biochemical parameters in ACS patients and the control group.

Variables	ACS patients		Non-ACS subjects	
	Correlation	*P* value	Correlation	*P* value
HDL (mmol/L)	−0.052	0.72	−0.012	0.936
LDL (mmol/L)	−0.23	0.108	0.007	0.962
FBS (mg/dl)	0.168	0.245	0.043	0.768
Creatinine (mg/dl)	0.147	0.31	−0.355	0.011*
Cholesterol (mg/dl)	−0.23	0.108	0.043	0.768
Triglyceride (mg/dl)	−0.003	0.983	−0.038	0.792
Na (meq/L)	−0.315	0.026*	−0.143	0.321
Ca (mg/dl)	0.144	0.319	0.013	0.93
K (meq/L)	0.125	0.385	−0.290	0.041*
*P* (mg/dl)	−0.271	0.057	−0.128	0.375
AST (U/L)	−0.200	0.164	0.074	0.612
ALT (U/L)	−0.187	0.193	0.097	0.503
ALP (U/L)	−0.062	0.670	0.001	0.993

* *p* < 0.05.

### 3.3 ANGPTL8 expression in patients and controls

**Figure 1:**
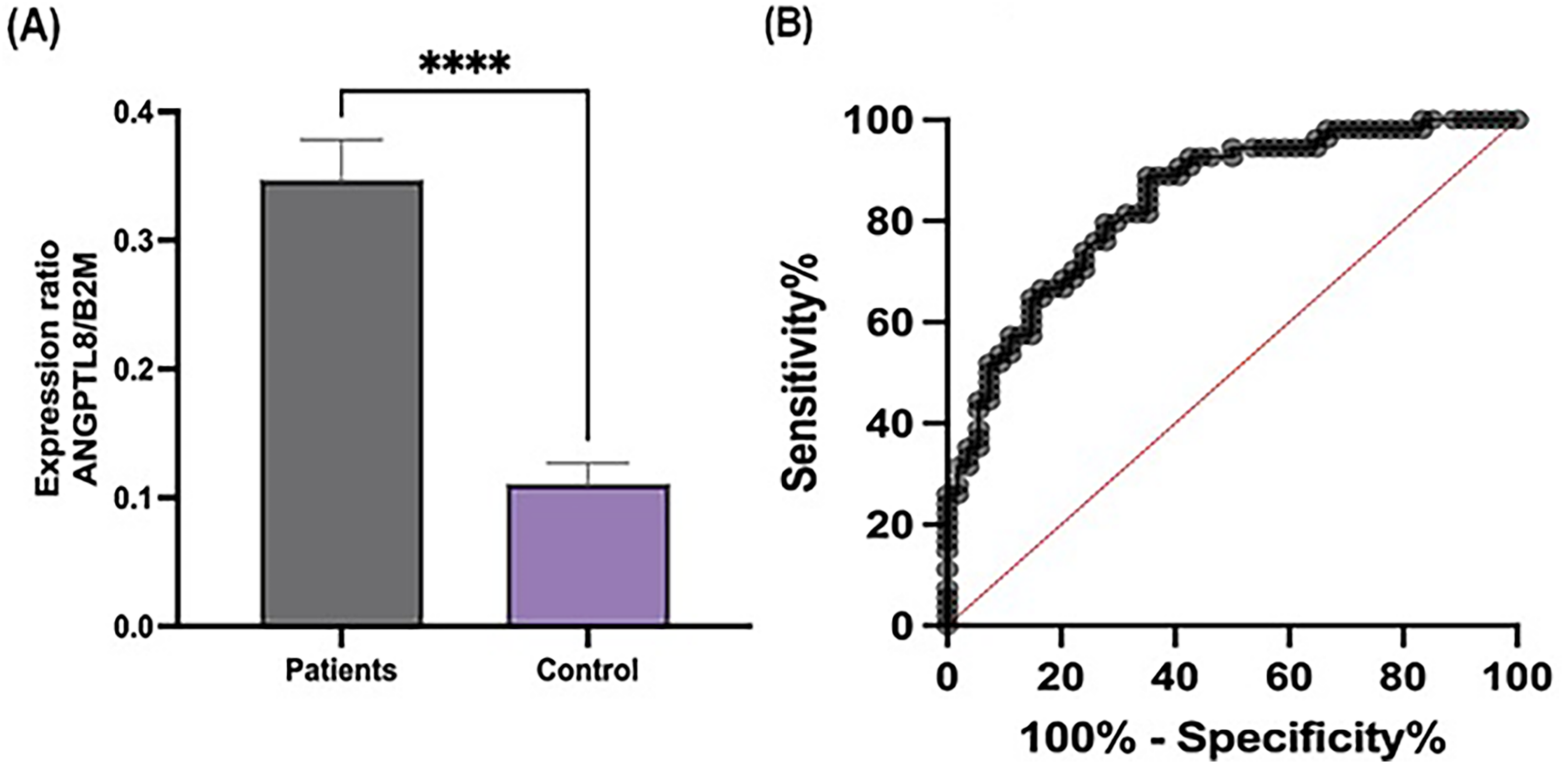
Expression levels and diagnostic performance of ANGPTL8 in patients and controls. **(A)** The expression ratio of ANGPTL8 to B2M was significantly higher in patients than in the controls. Data are presented as the mean ± SEM. ****indicates *p* < 0.0001. **(B)** ROC curve analysis of ANGPTL8 expression highlights its diagnostic potential. The curve demonstrated high sensitivity and specificity, supporting ANGPTL8 as a potential biomarker for distinguishing patients from controls.

The expression level of ANGPTL8 was significantly upregulated in patients with ACS compared to that in healthy controls (*P* < 0.001), as shown in Figure 1A. ROC curve analysis was performed to evaluate the diagnostic potential of ANGPTL8 expression. The analysis demonstrated that ANGPTL8 had moderate diagnostic ability for ACS, with an AUC of 0.83, sensitivity of 88%, and specificity of 64% (Figure 1B).

### 3.4 Correlations between ANGPTL8 expression and clinical characteristics

**Figure 2:**
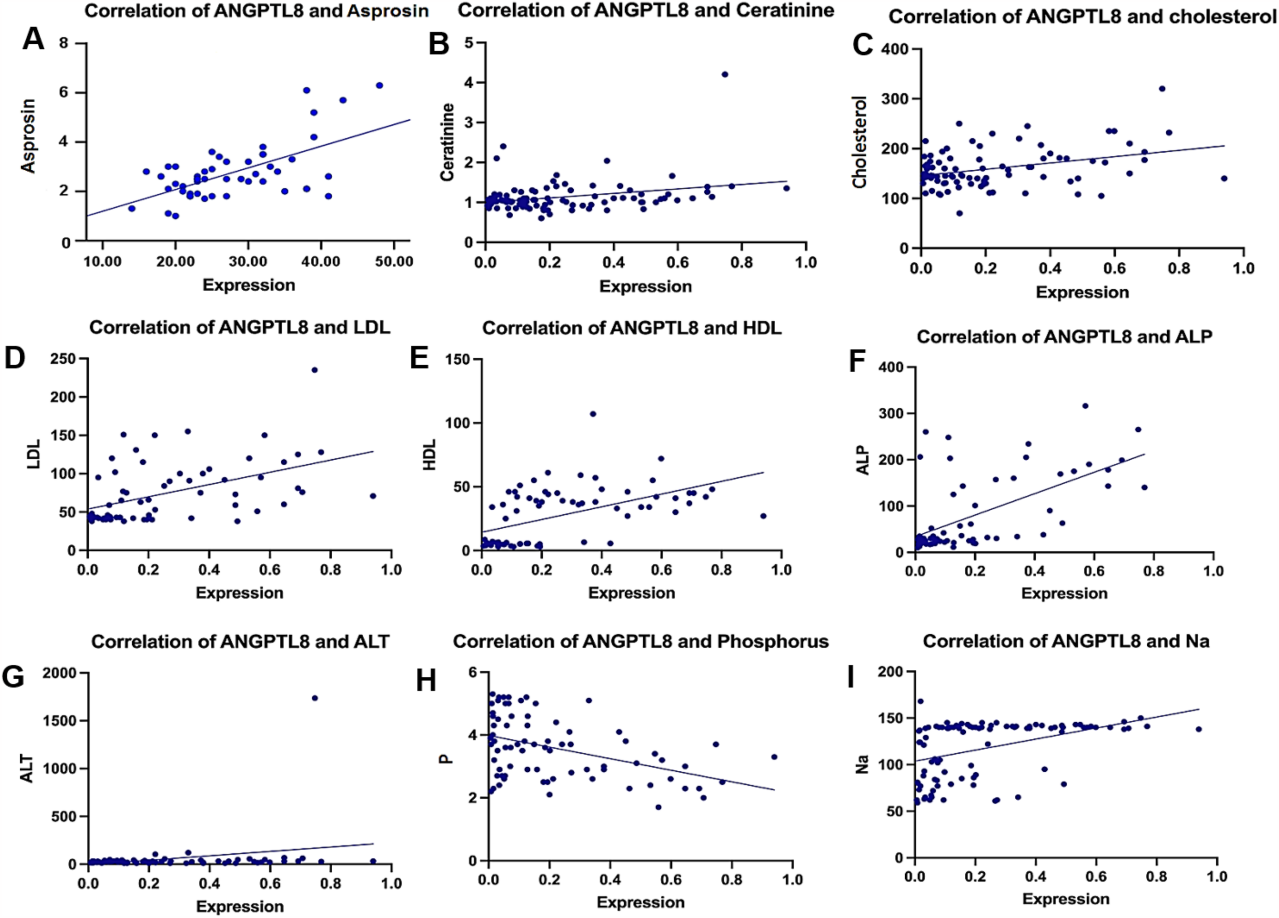
Correlation between ANGPTL8 expression and various clinical parameters. Scatter plots showing the correlation of ANGPTL8 levels with **(A)** asprosin, **(B)** creatinine, **(C)** cholesterol, **(D)** LDL, **(E)** HDL, **(F)** ALP, **(G)** ALT, **(H)** phosphorus, and **(I)** sodium levels. There were significant positive correlations between ANGPTL8 and asprosin (*r* = 0.795, *p* < 0.0001), HDL (*r* = 0.609, *p* < 0.0001), and ALP (*r* = 0.5857, *p* = 0.0001), as shown in the figure using various symbols. These findings suggest possible connections between ANGPTL8 expression and metabolic-inflammatory pathways in ACS.

ANGPTL8 expression decreased as phosphorus levels increased (−0.4015, *p* = 0.0003) and demonstrated an inverse pattern like that of potassium (−0.4585, *p* < 0.0001). By contrast, multiple positive associations emerged, varying in strength. The most substantial positive relationship was observed with asprosin concentration (0.795, *p* = 0.0001), while high-density lipoprotein (0.6090, *p* < 0.0001) and alkaline phosphatase (0.5857, *p* = 0.0001) showed moderately strong correlations. The analysis also identified significant positive relationships of decreasing magnitude with low-density lipoprotein (0.5281), sodium (0.4952), blood glucose (0.3719), creatinine (0.2849), alanine aminotransferase (0.2703), and total cholesterol (0.2167). Figure 2 shows the correlational patterns.

## 4 Discussion

Research outcomes reveal markedly increased blood asprosin concentrations and ANGPTL8 genetic activity in individuals experiencing ACS compared to healthy subjects, highlighting their potential as innovative diagnostic indicators. These findings illuminate their diagnostic value and possible mechanistic involvement in the metabolic-inflammatory cascades underlying ACS development. The diagnostic efficacy of asprosin (AUC = 0.95) and ANGPTL8 (AUC = 0.83) is remarkably encouraging. These indicators show comparable or enhanced detection capabilities relative to conventional markers such as troponins and CRP. While these findings support the potential clinical utility of Asprosin and ANGPTL8, it remains essential to determine whether their elevation can contribute to early ACS detection and improved patient management. Our findings showed that serum asprosin levels and ANGPTL8 gene expression were considerably higher in ACS patients than in controls. This suggests that these markers may be useful diagnostic markers. Our ROC analysis showed extremely high sensitivity and specificity for our markers (Asprosin: AUC = 0.95, sensitivity = 94%, specificity = 85%; ANGPTL8: AUC = 0.83, sensitivity = 88%, specificity = 64%). This suggests that these biomarkers will add to diagnostic precision when supplemented with traditional markers such as troponins. It is necessary to understand that this research was designed as a case-control study and not as a longitudinal study to screen for early ACS detection or assist in patient management.

Troponins are presently the best diagnostic tool for ACS, but they rise slowly after ischemic activity, making a diagnosis early challenging (14). Asprosin and ANGPTL8 may have the potential to diagnose ACS earlier because of their effects on metabolism and inflammation (15, 16). However, our study did not include serial measurements of biomarkers or long-term follow-ups of patients with these biomarkers, so it is not straightforward to determine if these markers continue to be useful predictors of outcomes outside the acute time frame. Future research needs to address this limitation by conducting studies that track biomarker changes over time and determine how they correlate with clinical outcomes. Additionally, incorporating these biomarkers into tests that utilize various methodologies alongside established cardiac markers can enhance the assessment and management of ACS risk.

Future studies should explore the correlation between asprosin, ANGPTL8, and cardiac troponins to determine whether these novel biomarkers offer complementary diagnostic value in ACS detection. While troponins remain the diagnostic benchmark for cardiac injury, their delayed elevation following an ischemic event limits early detection (17). Similarly, although CRP effectively indicates systemic inflammation, it lacks cardiovascular specificity (18). In this context, asprosin and ANGPTL8 could help bridge existing diagnostic gaps. Notably, asprosin's impressive sensitivity (94%) and specificity (85%) suggest its potential as an early metabolic indicator, while ANGPTL8's role in lipid regulation and arterial plaque formation offers distinct insights into ACS-related metabolic disturbances (13). The strong positive association between these markers (*r* = 0.795, *P* < 0.0001) indicates their collaborative role in the pathophysiology of ACS. Mechanistically, asprosin triggers hepatic glucose release and activates inflammatory pathways, particularly NF-κB signaling, which contributes to widespread inflammation and blood vessel dysfunction (10, 11). Concurrently, ANGPTL8 orchestrates lipid metabolism and influences immune cell activation, both of which are crucial for the progression of arterial plaque. This molecular synergy may accelerate plaque instability and damage to the heart muscle through amplified systemic inflammation and enhanced lipid accumulation (12, 13). In contrast to cardiac troponins, which are very specific for myocardial damage and rise late following ischemic insults (19), Asprosin and ANGPTL8 potentially have an advantage in terms of early detection of ACS. Although the gold standard remains troponins, they are not helpful in the setting of very slight myocardial damage or in patients presenting very early following symptom onset (20). Our results indicate that asprosin, with its metabolic-inflammatory phenotype, and ANGPTL8, a protein involved in lipid metabolism, may be complementary biomarkers for enhancing early diagnostic accuracy. Likewise, inflammatory biomarkers, such as CRP and IL-6, although helpful, are not ACS-specific, again underscoring the necessity for new biomarkers with greater diagnostic specificity (15). Further studies need to be done regarding the combined use of these markers in the clinical setting to enhance risk stratification models. Given their metabolic nature, several factors can influence these biomarkers, including fasting conditions, daily rhythms, and individual biological differences (8, 21). Another factor that could influence these markers is liver function, as previous studies have shown that asprosin and ANGPTL8 are affected by liver injury (22). However, in our study, there were no significant differences in liver function markers (ALT, AST, and ALP) between the ACS and control groups. This suggests that the liver did not significantly affect the biomarker level. Asprosin levels fluctuate with nutritional status, while ANGPTL8 responds to dietary patterns and cholesterol-lowering medications (23, 24).

Future investigations should evaluate these variables methodically to enhance their clinical reliability. The implications of this study significantly impact early detection and risk assessment strategies in ACS management. However, its practical implementation has several limitations that require further attention. One limitation of our study is the lack of time-course data to assess the peak concentrations and temporal fluctuations of asprosin and ANGPTL8 in patients with ACS. Serial sampling over multiple time points could provide a more comprehensive understanding of diagnostic kinetics. Future research should explore these temporal variations to determine the most clinically relevant timeframes for biomarker measurement and improve their predictive value for ACS diagnosis. Another limitation of our study is the absence of subgroup analysis, which could provide deeper insights into biomarker variations across different patient demographics, clinical presentations, or comorbidities. Future studies should incorporate subgroup analyses to better understand the potential differences in asprosin and ANGPTL8 expression based on factors such as age, sex, disease severity, or metabolic status. This approach would help to refine the diagnostic and prognostic utility of ACS management.

The development of standardized, cost-effective testing methods with quick results remains crucial. Furthermore, additional research must establish diagnostic thresholds for biological variability across different populations. Incorporating these novel markers into existing clinical protocols could enhance diagnostic precision. Combining them with traditional indicators may improve detection accuracy, particularly in early-stage or ambiguous cases. Validation through prospective trials is essential for confirming this approach. This investigation establishes a foundation for future research directions. Key areas include longitudinal studies examining the predictive value for adverse outcomes, mechanistic investigations exploring causal relationships, and large-scale multicenter trials across diverse populations. Additionally, evaluating the combined effectiveness of these novel and established markers in diagnostic algorithms could optimize clinical decision-making. Exploring the pharmaceutical modulation of these proteins may unlock new therapeutic possibilities. Moreover, understanding how lifestyle factors—such as smoking, exercise patterns, and concurrent medical conditions—affect these markers requires a detailed examination. The successful clinical integration of asprosin and ANGPTL8 measurements depends on developing practical, standardized testing protocols considering these various influencing factors. This study revealed distinct differences in the amounts of ANGPTL8 in ACS and healthy individuals, but not in the contrast groups. Larger groups of studies need to be conducted in the future to determine if factors such as health status, age, or medication have an impact on these biomarkers to better utilize them in the field of medicine.

## 5 Conclusion

This study emphasizes the pronounced increase in serum asprosin concentrations and ANGPTL8 gene expression in patients with ACS and their diagnostic and prognostic value. The positive synergism between the given biomarkers indicated their joint involvement in the metabolic and inflammatory pathways of ACS. In addition, biomarker expression differences specific to subgroups need to be validated in larger, more diverse populations to see if demographic or clinical variables influence their diagnostic usefulness.Further studies are needed to confirm these results and investigate their therapeutic implications for ACS outcomes improvement.

## Data Availability

The raw data supporting the conclusions of this article will be made available by the authors, without undue reservation.
